# Acceptability of a chikungunya vaccine and dengue vaccine among travelers in Martinique (French West Indies), for the travel and for their home territory

**DOI:** 10.1016/j.ijregi.2025.100791

**Published:** 2025-10-24

**Authors:** Fanny Quenard, Francis Pecout, Thierno A Barry, Romain Mortier, Marie-Paule Ferdinand, Ornella Cabras, André Cabie

**Affiliations:** 1 Travel clinic in the Infectious and Tropical Diseases Unit, Martinique University Hospital, Fort de France, Martinique, France; 2 AEROVAC Travel clinic, Martinique, France; 3 PCCEI, Montpellier University, Antilles University, INSERM, EFS, Montpellier, France

**Keywords:** Vaccine acceptability, Dengue vaccine, Chikungunya vaccine, Martinique

## Abstract

**Objectives:**

The incidence of chikungunya and dengue is increasing worldwide. Vaccines are now available against these two arboviral diseases.

**Methods:**

We evaluated the acceptability of live chikungunya and dengue vaccines (VLA1553 and TAK-003) among users of Martinique’s travel clinics from January to April 2025. We asked whether they would want these vaccines for travel to areas with exposure risk and/or for their home territory.

**Results:**

While planning travel, 41% of respondents expressed interest in the chikungunya vaccine, and 55% were interested in the dengue vaccine. For the Martinique territory, 55% were willing to receive the chikungunya vaccine, and 58% were willing to receive the dengue vaccine.

**Conclusions:**

In both contexts (travel and home territory), acceptability was higher for dengue than for chikungunya vaccination. Acceptability for both vaccines was also higher for the home territory than for travel to areas with exposure risk. Few data are available on chikungunya vaccine acceptability. We found similar acceptability for this vaccine in Martinique as reported in the United States Virgin Islands (56%), an overseas territory of the United States in the Caribbean, and in La Réunion, another French overseas territory in the Indian Ocean (60.5% in the hypothetical full-reimbursement scenario).

## Highlights

• We evaluated the acceptance of chikungunya and dengue live vaccines.
• Our study focused on users of Martinique’s travel clinics (French West Indies).
• The population surveyed is regularly exposed to arboviral diseases.
• Participants were more interested in vaccination for Martinique than for a travel.
• Participants were more interested in dengue vaccination.

## Introduction

Chikungunya and dengue are arboviral diseases transmitted by *Aedes aegypti* and *Aedes albopictus* mosquitoes and are spreading worldwide. Between January and August 2025, the European Centre for Disease Prevention and Control reported 317,000 chikungunya cases globally, and in 2024, 15 million dengue cases were recorded.

Chikungunya causes an acute febrile illness with polyarthralgia; its burden is mainly due to a chronic phase that occurs in 20-60% of cases [1]. Dengue progresses through three phases—febrile, critical, and recovery—and its morbidity and mortality are linked to its severe forms (2-4%).

Martinique, a French overseas island in the Caribbean with 355,500 inhabitants, where *Aedes aegypti* is present year-round, experienced a major chikungunya outbreak in 2014, resulting in 1196 hospitalizations and a post-epidemic seroprevalence of 43.2% [2]. Dengue is both endemic and epidemic in Martinique; the 2023-2024 outbreak caused 23,319 medical consultations, 30 severe cases, and nine deaths [3].

Frequent travel between Martinique and mainland France facilitates the importation and exportation of arboviral infections. The population of Martinique is aging, and older age is a known risk factor for severe dengue and chikungunya. Overall vaccination acceptance in Martinique was estimated at 59% in 2021 [4].

Until recently, prevention relied solely on vector control and personal protection. The European Medicines Agency has now approved live vaccines against chikungunya (VLA1553, June 2024) and dengue (TAK-003, December 2022). This study evaluates the acceptability of these vaccines among travelers living in Martinique.

## Materials and methods

This prospective study assessed the acceptability of the chikungunya (VLA1553) and dengue (TAK-003) vaccines among adults attending two travel medicine clinics in Martinique.

Participants voluntarily completed an anonymous paper questionnaire between January 23 and April 15, 2025 (see Supplementary Material). At that time, no French recommendations existed for these vaccines in travelers. The United States Centers for Disease Control and Prevention guidelines (January 2025) were used as a reference (see Supplementary Material).

The primary objective was to estimate the proportion of participants willing to receive the chikungunya and/or dengue vaccine for travel to areas at risk of exposure, both if the vaccine were free and if it required payment. A secondary objective was to evaluate willingness to be vaccinated if the vaccine were available and reimbursed for residents of Martinique. Age and travel characteristics were also collected.

This study did not involve human experimentation and received approval from the National Ethics Committee for Research in Infectious Diseases (IRB No. 00011642). Statistical analyses were performed using STATA, with descriptive statistics expressed as frequencies and percentages.

## Results

A total of 244 participants were included, with a median age of 50 years; 48 participants (20%) were aged ≥65 years. The most common destinations were French Guiana (50%), West Africa (25%), and Brazil (9%). The median trip duration was 10 days, and the median interval between consultation and departure was 20 days; only 24% consulted more than 38 days before travel.

According to the Centers for Disease Control and Prevention criteria, 26 participants planned travel to areas with chikungunya risk and 30 to areas with dengue risk. For travel purposes, 41% (88 out of 216 respondents) were interested in the chikungunya vaccine, and an additional 32% of those initially uninterested would accept it if covered by health insurance. Similarly, 55% (117 out of 213) expressed interest in the dengue vaccine for travel, and another 12% would accept it if reimbursed.

If vaccines were available for residents of Martinique, 55% (118 out of 213) would be willing to receive the chikungunya vaccine and 58% (121 out of 210) the dengue vaccine. A prior history of chikungunya was reported by 28% of respondents; acceptance of the chikungunya vaccine (55%) did not differ by past infection.

## Discussion

This population is regularly exposed to arboviral infections. Vaccine acceptance was higher for dengue (58%) than for chikungunya, and participants were more inclined to vaccinate for their home territory than for travel, reflecting a greater perceived risk at home.

Comparable findings were observed in the United States Virgin Islands in 2014, where 56% of residents expressed willingness to receive a hypothetical chikungunya vaccine [5]. Similar results were reported in La Réunion at the onset of the 2025 chikungunya outbreak, with 60.5% acceptance before a vaccination campaign [6].

A 2023 meta-analysis reported a global dengue vaccine acceptance rate of 88.3% (95% confidence interval 81.0-93.0) [7]. Practical constraints for travelers include timing: only 24% of participants consulted early enough (≥38 days before departure) to accommodate vaccination schedules—particularly relevant for TAK-003, which requires two doses administered three months apart.

A 2024 modeling study in Paraguay estimated that initiating a chikungunya vaccination campaign early in an outbreak, achieving 40% coverage and 75% efficacy, could prevent 23% of cases and deaths [8]. For dengue, the World Health Organization emphasizes the greatest benefit from integrating vaccination into routine immunization programs [9].

This first study of arboviral vaccine acceptability in Martinique does not represent the general population. Participants were self-selected travelers, typically older and of higher socioeconomic status. The study concluded before pharmacovigilance alerts on VLA1553 use in individuals aged over 65 years. Another chikungunya vaccine, based on virus-like particles (CHIK-VLP), has since received a positive European Medicines Agency opinion.

## Conclusion

This first assessment of chikungunya and dengue vaccine acceptability among travelers from Martinique found greater willingness to vaccinate for local protection than for travel, and higher acceptance for dengue than for chikungunya.

Perceived disease severity appears to influence acceptance—participants likely view dengue as more serious. Further studies in the general population are needed to identify determinants of vaccine acceptance and to inform vaccination strategies in the French Caribbean.

## Declaration of competing interest

The authors declare no competing interests.

## Appendix Supplementary materials

Image, application 1

Image, application 2
