# Supplementary material for: Acceptability of a chikungunya vaccine and dengue vaccine among travelers in Martinique (French West Indies), for the travel and for their home territory
Source: IJID Reg. 2025 Oct 24;17:100791. doi: 10.1016/j.ijregi.2025.100791 (PMC12664037; doi:10.1016/j.ijregi.2025.100791)
Supplement: Supplementary file 1 [file mmc1.docx]

CDC criteria for risk of exposure to chikungunya are:
• When visiting a country in the epidemic phase

• When visiting a country or territory without an outbreak but with human transmission of the chikungunya virus in the past 5 years and:

o People aged 65 and over, particularly those with underlying medical conditions and likely to have at least two cumulative weeks of exposure to mosquitoes OR

o People staying for a cumulative period of 6 months or more in a risk area.

CDC criteria for risk of exposure to dengue is visiting a country with higher-than-expected numbers of dengue fever cases.
