# Supplementary material for: Acceptability of a chikungunya vaccine and dengue vaccine among travelers in Martinique (French West Indies), for the travel and for their home territory
Source: IJID Reg. 2025 Oct 24;17:100791. doi: 10.1016/j.ijregi.2025.100791 (PMC12664037; doi:10.1016/j.ijregi.2025.100791)
Supplement: Supplementary file 2 [file mmc2.docx]

QUESTIONNAIRE ANONYME

Un vaccin vivant contre le chikungunya (1) (produit à partir du virus vivant atténué, processus similaire à celui contre la fièvre jaune) a été récemment accepté par les autorités de santé européennes (EMA(2)) permettant sa mise sur le marché en France, au prix d’environ 195€ sans remboursement actuellement car les avis de la Haute Autorité de Santé sont en attente.

Ce vaccin étant disponible et déjà utilisé dans certains pays, nous souhaiterions avoir votre avis concernant ce vaccin.

Le vaccin IXCHIQ a montré une efficacité dans les études de phase 3 sur des corrélats de protection sur la réponse humorale (taux d’anticorps). Il n’y a pas pour le moment de résultats d’efficacité vaccinale par des essais cliniques contrôlés randomisés dans des régions d’épidémie.

Aux Etats-Unis, le CDC(3) le recommandent pour les voyageurs de plus de 18 ans, en absence de contre-indication :

- **En cas de séjour dans un pays en phase épidémique** (carte 1)
- En cas de **séjour dans un pays ou un territoire** sans épidémie mais **avec une transmission** du virus du chikungunya chez l'homme **au cours des 5 dernières années** (carte 2) pour :
  - les personnes âgées de **65 ans et plus**, en particulier celles qui présentent des maladies sous-jacentes et qui sont susceptibles d'avoir une **exposition d’au moins deux semaines cumulées** aux moustiques ;
  - ou les personnes séjournant pendant une **période cumulée de 6 mois ou plus** en zone à risque

Carte 1 : Pays ou zones en phase épidémique en décembre 2024 :


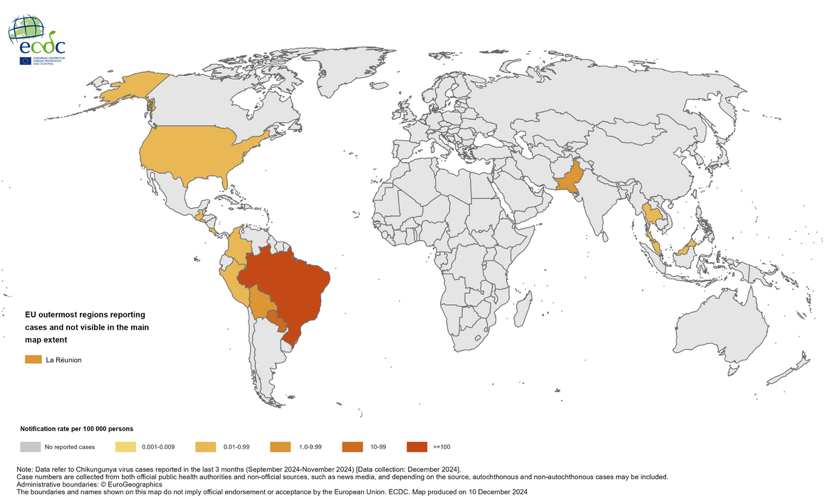


Carte 2 : Pays ou zones où il y a eu circulation du virus dans les 5 dernières années :


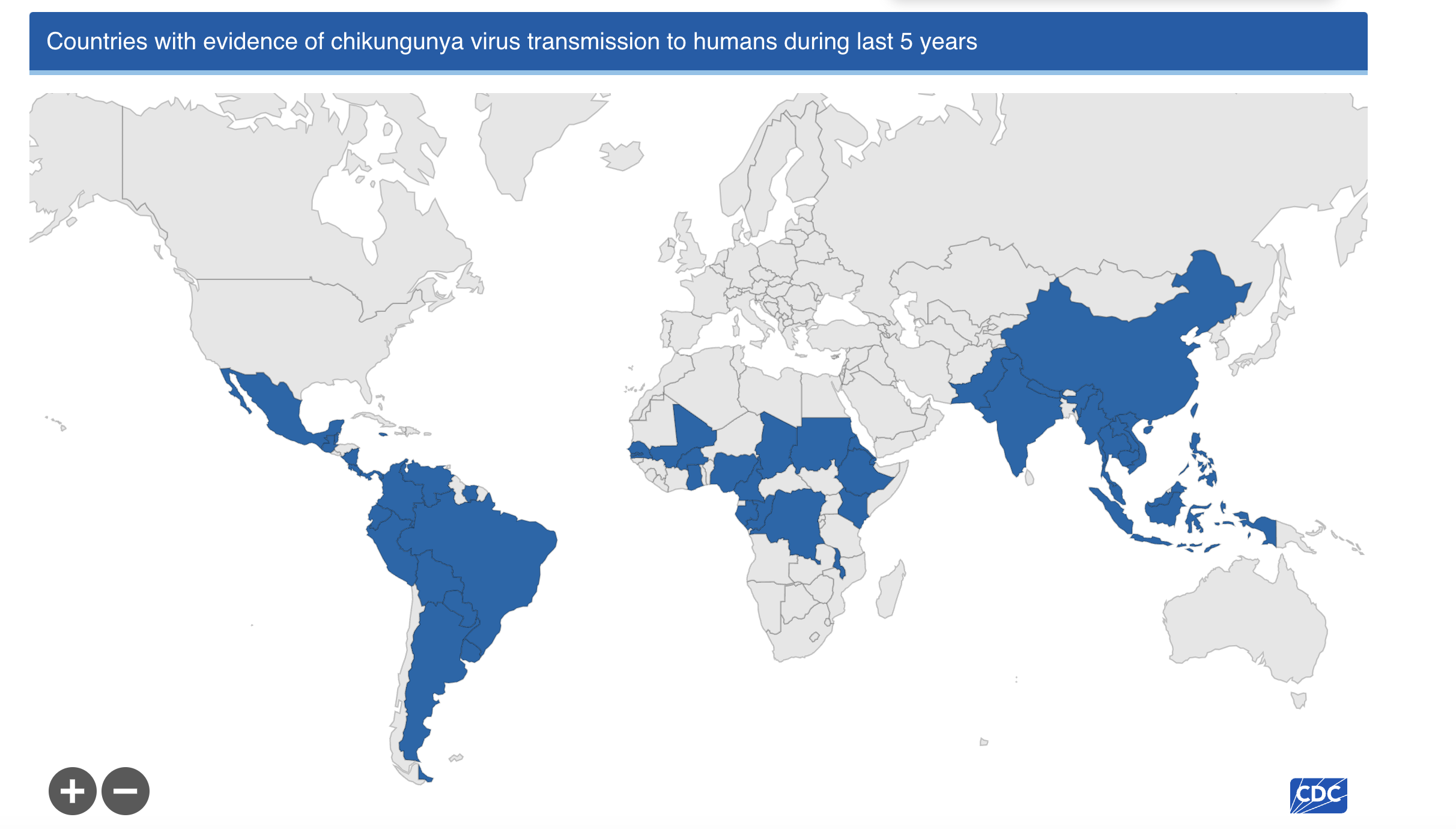


Ce vaccin vivant ne peut actuellement être fait à un intervalle de moins de 28 jours avec celui de la fièvre jaune.

Les effets indésirables potentiels sont : au site d’injection : sensibilité au toucher (10,8 %) et douleur (6,1 %). Les effets indésirables systémiques les plus fréquents étaient : céphalées (32 %), fatigue (29,4 %), myalgie (23,7 %), arthralgie (16,6 %), fièvre (13,8 %) et nausées (11,4 %).

Les contre-indications sont allergie à un des composants, immunodépression en raison d’une maladie ou d’un traitement médical (par ex. à la suite d’un cancer hématologique ou d’une tumeur solide, de l’administration d’une chimiothérapie, d’une immunodéficience congénitale ou d’un traitement immunosuppresseur à long terme, ou les patients infectés par le VIH sévèrement immunodéprimés). La grossesse et l’allaitement sont peu étudiées et nécessite une discussion autour des risques et bénéfices attendus.

Questionnaire Acceptabilité Vaccin IXCHIQ®

1. Quel est votre âge ?
2. Avez-vous déjà eu le chikungunya ?
3. Où voyagez-vous ?
4. Dans combien de jours partez-vous ?
5. Pour combien de temps ?
6. Seriez-vous intéressé pour effectuer cette vaccination dans le cadre d’un voyage en zone d’endémie avec probabilité d’exposition au chikungunya ?
7. Seriez-vous intéressé pour effectuer cette vaccination si elle était remboursée pour les personnes vivantes en Martinique ?
8. Seriez-vous intéressé pour effectuer cette vaccination si elle était remboursée dans le cadre d’un voyage en zone d’endémie avec probabilité d’exposition au chikungunya ?

L’agence Européenne du Médicament a également autorisé un vaccin vivant atténué, quadrivalent contre les quatre sérotypes (1, 2, 3 et 4) du virus de la **dengue.** Ce vaccin n’est pas encore en vente en France et les recommandations concernant ce vaccin sont en cours.

1. Seriez-vous intéressé pour effectuer cette vaccination dans le cadre d’un voyage en zone d’endémie avec probabilité d’exposition à la dengue ?
2. Seriez-vous intéressé pour effectuer cette vaccination contre la dengue si elle était remboursée pour les personnes vivantes en Martinique ?
3. Seriez-vous intéressé pour effectuer cette vaccination si elle était remboursée dans le cadre d’un voyage en zone d’endémie avec probabilité d’exposition à la dengue ?

1. Ly H. Ixchiq (VLA1553): The first FDA-approved vaccine to prevent disease caused by Chikungunya virus infection. Vol. 15, Virulence. Taylor and Francis Ltd.; 2024.

2. Ixchiq | European Medicines Agency (EMA) [Internet]. [cited 2024 Dec 4]. Available from: https://www.ema.europa.eu/en/medicines/human/EPAR/ixchiq

3. Chikungunya Vaccine Information for Healthcare Providers | Chikungunya Virus | CDC [Internet]. [cited 2024 Dec 4]. Available from: https://www.cdc.gov/chikungunya/hcp/vaccine/index.html
